# Effect of Dietary Supplementation of Different Fiber Levels Obtained From Tucumã Peel on Zootechnical and Hematological Performance in Juvenile Tambaqui (*Colossoma macropomum*)

**DOI:** 10.1155/anu/1478631

**Published:** 2026-09-03

**Authors:** Alcilany Nascimento Paiva, Wallice Paxiuba Duncan, Jaime Paiva Lopes Aguiar, Francisca das Chagas do Amaral Souza

**Affiliations:** ^1^ Postgraduate Program in Agriculture in the Humid Tropics, National Institute for Amazonian Research (INPA), Manaus 69067–375, Amazonas, Brazil, inpa.gov.br; ^2^ Laboratory of Functional Morphology, Federal University of Amazonas (UFAM), Manaus 69077–000, Amazonas, Brazil, ufam.edu.br; ^3^ Laboratory of Functional Analysis and Food Chemistry, National Institute for Amazonian Research (INPA), Manaus 69067–375, Amazonas, Brazil, inpa.gov.br

**Keywords:** agro-industrial by-products, fish physiology, functional ingredients, lipid metabolism, sustainable aquaculture

## Abstract

The present study evaluated the effects of dietary inclusion of fiber derived from tucumã peel (*Astrocaryum aculeatum*) on the zootechnical performance and physiological responses of juvenile tambaqui (*Colossoma macropomum*). Five approximately isonitrogenous and isoenergetic diets were formulated with increasing levels of tucumã peel (0%, 3%, 6%, 9%, and 12%). A total of 225 fish were distributed in a completely randomized design with three replicates per treatment. Ten fish per experimental unit were individually identified using microchips to allow accurate monitoring of individual growth while minimizing handling stress. After 45 days, growth performance, hematological parameters, and plasma biochemical indicators were evaluated. Dietary fiber inclusion did not affect growth performance or hematological parameters, which remained within normal physiological ranges. However, increasing fiber levels significantly reduced plasma cholesterol and triglyceride concentrations, suggesting a modulatory effect on lipid metabolism. The results indicate that tucumã peel can be included as a dietary fiber source for tambaqui without adverse effects on performance under the conditions tested, although further studies are needed to determine optimal inclusion levels and long‐term effects.

**Summary**

• Tucumã peel fiber was evaluated as a dietary ingredient for juvenile tambaqui.
• Fiber inclusion up to 12% did not impair the growth performance or feed efficiency.
• Hematological parameters remained within normal physiological ranges.
• Higher fiber levels reduced plasma cholesterol and triglycerides.
• Tucumã peel is a sustainable alternative ingredient for tambaqui diets.

## 1. Introduction

The use of agro‐industrial by‐products as raw materials or substitute ingredients in human and/or animal diets has become an extremely relevant alternative since the amount of nutrients present in these by‐products is similar to or greater than those found in the main raw material [1]. Among the fruits produced in the Amazon region is tucumã (*Astrocaryum aculeatum*), which is highly exploited economically for its fresh pulp and other by‐products that are used for consumption [2]. According to Machado et al. [3], tucumã the peel is generally discarded during fruit processing, although it represents a potential alternative source of dietary fiber for tambaqui feeds, contributing to the valorization of this agro‐industrial by‐product. The chemical composition of tucumã peel determined in the present study was 55.9% crude fiber, 9.3% protein, 24.9% lipids, and 6.7% carbohydrates. Dietary fiber plays an important role in the nutrition of omnivorous fish, contributing to digestive physiology and intestinal function [4]. Dietary fiber consists mainly of nondigestible polysaccharides that may be partially fermented by the intestinal microbiota, depending on the fiber fraction and fish species. Soluble and insoluble fibers may exert distinct physiological and metabolic effects, contributing to digestive function and intestinal health, although studies on dietary fiber inclusion in fish diets remain limited. Tambaqui (*Colossoma macropomum*) is an omnivorous Neotropical fish species of great economic importance to Brazilian aquaculture and is one of the main native species cultivated in the country. Adequate dietary fiber intake is essential for digestive physiology and the excretion of nitrogenous waste in omnivorous fish, including tambaqui [5]. Abreu et al. [6] reported that since it is an omnivorous fish, its food in the natural environment consists mainly of zooplankton, fruits, and peel; therefore, it is an animal that has high levels of fiber in its diet, which justifies supplementing the feed of farmed fish with fibers obtained from the tucumã peel.

Despite the increasing use of plant‐based ingredients in aquafeeds, there is still limited information regarding the physiological effects of dietary fiber derived from Amazonian agro‐industrial by‐products in Neotropical fish species, particularly tambaqui (*Colossoma macropomum*). Furthermore, the effects of different dietary fiber inclusion levels on lipid metabolism and hematological responses remain poorly understood.

Therefore, we hypothesized that dietary inclusion of tucumã peel, as a source of dietary fiber and bioactive compounds, could be incorporated into tambaqui diets without impairing zootechnical performance while promoting metabolic and hematological modulation.

## 2. Materials and Methods

### 2.1. Obtaining and Acclimating the Fish

A total of 225 fish from a fish farm located on the Manuel Urbano road (AM‐070) were used. The animals were acclimated in a 1000 L water tank with continuous aeration and water flow. For food conditioning, commercial feed with 40% crude protein (CP) was used, supplied twice a day at 8:00 and 17:00 until apparent satiety. All experimental protocols were approved by the Ethics Committee on the Use of Animals (CEUA) of the Federal University of Amazonas, under No. 007/2018‐CEUA/UFAM.

### 2.2. Feed Preparation

The tucumã peel (*Astrocaryum aculeatum*) was purchased from local tucumã pulp traders and sent to the Food Physics–Chemistry Laboratory (LFQA) of the National Research Institute of the Amazon‐INPA for cleaning. It was initially washed in running water and sanitized with a 200 ppm hypochlorite solution and dried in an oven with air circulation for 24 h at 60°C. The samples were then crushed, and bromatological analyses (Table 1) were performed according to the methodology proposed by IAL [7] for moisture, CP, lipids, and ash; for the insoluble and soluble dietary fiber contents, the method of Asp et al. [8] was followed. The results obtained for the proximal composition of the ingredients to be used in the formulations were determined according to the methodology described by Guimarães et al. [9] and Rostagno [10]. Five isoproteic and isocaloric pellet diets were prepared as shown in Table 1. The diets were composed of 30% CP and increasing levels of tucumã peel, *Astrocaryum aculeatum* (0%, 3%, 6%, 9%, and 12%), as the main source of fiber.

**Table 1:** Ingredients and calculated composition of experimental diets containing different levels of tucumã peel for juvenile tambaqui (*Colossoma macropomum*).

Ingredient (%)	T0	T1	T2	T3	T4
Soybean meal	47.81	48.58	49.34	50.11	50.88
Corn	23.91	22.02	20.13	18.24	16.34
Fish meal	7.00	7.00	7.00	7.00	7.00
Wheat bran	16.00	13.00	10.00	7.00	4.00
Tucumã peel	0.00	3.30	6.00	9.00	12.00
DL‐methionine	0.77	0.77	0.77	0.77	0.77
Soybean oil	2.00	3.13	4.25	5.38	6.50
Dicalcium phosphate	1.39	1.39	1.39	1.39	1.39
Limestone	0.52	0.52	0.52	0.52	0.52
Salt	0.10	0.10	0.10	0.10	0.10
Vitamin–mineral premix^a^	0.50	0.50	0.50	0.50	0.50

Calculated composition	T0	T1	T2	T3	T4
Gross energy (MJ kg^−1^)	3.36	3.36	3.36	3.36	3.36
Digestible protein (%)	26.52	26.35	26.18	26.01	25.84
Crude protein (%)	30.00	30.00	30.00	30.00	30.00
Crude fiber (%)	3.50	4.96	6.08	7.86	9.31
Ether extract (%)	4.74	6.43	8.13	9.82	11.52
Digestible energy/digestible protein ratio	114.05	114.02	113.99	113.96	113.94
Starch (%)	25.45	23.51	21.57	19.63	17.69

*Note:* Diets were formulated to be isonitrogenous and isoenergetic.

^a^Vitamin–mineral premix composition (per kg of diet): vitamin A, 10,000 IU; vitamin D_3_, 2000 IU; vitamin E, 100 mg; vitamin K_3_, 10 mg; vitamin B_1_, 10 mg; vitamin B_2_, 20 mg; vitamin B_6_, 15 mg; vitamin B_12_, 0.05 mg; niacin, 100 mg; folic acid, 5 mg; calcium pantothenate, 50 mg; biotin, 0.5 mg; choline chloride, 500 mg; iron, 50 mg; zinc, 50 mg; manganese, 30 mg; copper, 10 mg; iodine, 1 mg; selenium, 0.3 mg.

### 2.3. Conduction and Experimental Design

The experiment was conducted at the Federal University of Amazonas, located in Manaus, Amazonas, Brazil. A completely randomized design was used with five treatments and three replicates over a period of 45 days. Fifteen circular tanks (310 L) were used, and 15 fish were stocked per experimental unit. Of these, 10 fish were individually identified using microchips (ISO FDX‐B 134.2 kHz transponder) to allow accurate monitoring of individual growth while minimizing handling stress. A total of 225 fish were used, with an initial mean weight of 23.29 ± 15.02 g. Fish were fed twice daily (08:00 and 17:00) to apparent satiety.

Water quality parameters were monitored daily and included dissolved oxygen, temperature, pH, ammonia, and dissolved minerals (sodium, potassium, and calcium). The dissolved oxygen and temperature were measured using a Hanna HI9147 portable oximeter. The pH was measured using a pHTek PH‐8B bench pH meter. Ammonia was quantified by the Nessler method using a Thermo Scientific Multiscan Go microplate spectrophotometer. Dissolved sodium, potassium, and calcium ions were analyzed using flame photometry (Digimed DM62 flame photometer).

### 2.4. Evaluation of Zootechnical Performance

Every 15 days throughout the experimental period, standard length and body weight were measured, totaling four measurements per fish. Fish were briefly anesthetized with benzocaine (0.5 mg L^−1^), and biometrics were performed using a semianalytical balance and a measuring ruler.

Based on these data, the following zootechnical performance parameters were calculated:

Weight gainWG= Final mean weightg− initial mean weightg,

Specific growth RateSGR=100×lnfinal weight− lninitial weight/ experimental perioddays,

Relative weight gainRWG=100×final weight− initial weight/ initial weight,

feed‐related growth rate, length gain, and growth rate.

### 2.5. Evaluation of Hematological Parameters

At the end of the experimental period, blood samples were collected from 10 fish per experimental unit for hematological and biochemical analyses. Fish were fasted for 24 h prior to the sampling. Blood was collected from the caudal vein using a 3 mL syringe containing heparin as an anticoagulant and immediately subjected to hematological analysis.

Hematological parameters included hematocrit (Ht), determined by the microhematocrit method, and hemoglobin (Hb) concentration, determined by the cyanmethemoglobin method. Circulating erythrocytes (RBCs) were counted using a Neubauer chamber after dilution in a formaldehyde‐citrate solution. Based on these parameters, Wintrobe hematimetric indices were calculated: mean corpuscular volume (MCV), mean corpuscular hemoglobin (MCH), and MCH concentration (MCHC).

For biochemical analysis, blood samples were centrifuged at 10,000 rpm for 10 min, and plasma was used to determine glucose (glucose oxidase method), total protein (Biuret method), albumin (bromocresol green method), triglycerides, and total cholesterol (enzymatic Trinder method). All analyses were performed in duplicate using commercial kits (Labtest Diagnóstica S.A., Brazil), and absorbance was measured using a Thermo Scientific Multiscan Go microplate spectrophotometer.

### 2.6. Fatty Acid Evaluation

Muscle samples were collected from 10 fish per experimental unit and homogenized. Total lipids were extracted and determined according to the methodology proposed by Bligh and Dyer [11]. The fatty acids were converted into fatty acid methyl esters (FAMEs) and analyzed using gas chromatography (Shimadzu GC‐2010 Plus, Kyoto, Japan) equipped with a flame ionization detector.

Separation was performed on a 30 m RTxR‐5 capillary‐fused silica column (0.25 mm internal diameter, 0.25 µm film thickness). The operating conditions were as follows: programmed oven temperature from 80 to 220°C at 5°C/min; injector temperature, 230°C; detector temperature, 240°C; carrier gas, hydrogen; linear gas velocity, 40 cm/s; and a split ratio of 1:50.

Fatty acids were identified by comparing retention times with known standards (Sigma Supelco) and quantified by normalization of peak areas.

### 2.7. Statistical Analysis

Data were tested for normality using the Kolmogorov–Smirnov test and for homogeneity of variances using Levene’s test. Subsequently, data were subjected to one‐way analysis of variance (ANOVA). When significant differences were detected, means were compared using Tukey’s post hoc test. In cases where assumptions were not met, the Kruskal–Wallis test was applied. Statistical analyses were performed using Minitab version 18.

## 3. Results

The tucumã peel used in the formulations showed the following composition: 55.97% crude fiber, 9.36% CP, 24.91% ether extract, 6.77% carbohydrates, 2.71% ash, and 0.27% moisture.

The analyzed water quality parameters remained stable throughout the experimental period. Over the 45 days of the experiment, dissolved oxygen levels ranged from 4 to 6 mg L^−1^, water temperature remained ~28.5°C, and pH ranged from 4.6 to 5.5. Dissolved salts and ammonia remained constant at low levels.

The increase in dietary fiber levels from tucumã peel (*Astrocaryum aculeatum*) did not affect zootechnical performance, hematological parameters, glucose levels, total protein, or albumin in tambaqui (*Colossoma macropomum*). However, a greater supply of fiber from tucumã peel in the diet significantly reduced plasma cholesterol and triglyceride levels.

Although studies on dietary fiber in fish are still limited, according to Fracalossi et al. [4], dietary fiber consists mainly of nondigestible polysaccharides that may be partially fermented by intestinal microbiota, depending on the fiber fraction and fish species. Soluble and insoluble fibers may exert different physiological and metabolic effects, contributing to the digestive function and intestinal health.

In this study, it was verified that the different levels of fiber present in tucumã peel (*Astrocaryum aculeatum*) did not affect biomass gain, specific growth rate, weight gain rate, relative weight gain, length gain, or growth rate, as shown in Table 2, with zero mortality observed in all treatments.

**Table 2:** Mean ± standard deviation of zootechnical performance of tambaqui (*Colossoma macropomum*) juveniles fed different levels of tucumã peel (*Astrocaryum aculeatum*).

Zootechnical parameters	0%	3%	6%	9%	12%
WG (g)	20.5ᵇ ± 6.6	23.7ᵃ ± 8.2	22.6ᵃ ± 9.8	22.1ᵃ ± 9.4	23.3ᵃ ± 11.9
SGR (%)	1.6ᵃ ± 0.6	1.6ᵃ ± 0.5	1.6ᵃ ± 0.6	1.7ᵃ ± 0.6	1.5ᵃ ± 0.7
WGR (%)	47.0ᵃ ± 13.0	47.3ᵃ ± 11.0	47.0ᵃ ± 12.8	48.4ᵃ ± 13.2	44.6ᵃ ± 15.4
RWG (%)	99.9ᵃ ± 48.0	98.1ᵃ ± 40.0	100.3ᵃ ± 49.9	106.3ᵃ ± 50.9	97.4ᵃ ± 64.3
LG (cm)	2.5ᵃ ± 0.7	2.5ᵃ ± 0.6	2.4ᵃ ± 0.6	2.5ᵃ ± 0.7	2.3ᵃ ± 0.9
GR (%)	22.4ᵃ ± 6.8	21.8ᵃ ± 6.0	21.6ᵃ ± 6.3	22.6ᵃ ± 6.5	20.4ᵃ ± 7.3

*Note:* Means followed by the same letter in the same row do not differ significantly (*p*  > 0.05; ANOVA followed by Tukey’s test).

Abbreviations: GR, growth rate; LG, length gain; RWG, relative weight gain; SGR, specific growth rate; WG, weight gain; WGR, weight gain rate.

In zootechnical performance evaluation, growth is one of the most commonly used variables to assess the response of a given fish species to experimental ingredients as it is more practical and sensitive to the adequacy of nutrient and energy levels. It is also a predominant variable in production systems as it has a direct relationship with productivity and profitability. Growth can be measured by weight gain or growth rate over time, such as the specific growth rate [4].

In the present study, growth was not affected by the inclusion of tucumã peel (*Astrocaryum aculeatum*) in the formulated diets, likely because the diets were isoenergetic and isoproteic, varying only in the fiber content. Although the diets were formulated to be approximately isoproteic and isoenergetic, the inclusion of tucumã peel may have altered nutrient digestibility and biologically available energy due to its fiber content and physicochemical characteristics. Therefore, the metabolic responses observed in tambaqui juveniles may also be associated with differences in nutrient utilization efficiency. Similar results were reported by Lemos et al. [12], who evaluated coconut bran in diets for tambaqui (*Colossoma macropomum*), and by Lopes et al. [13], who demonstrated that babassu bran levels ranging from 0% to 12% in tambaqui diets also do not affect the zootechnical performance in this species. Fabregat et al. [14] evaluated different levels of fiber from various sources (soybean hull, soybean meal, sunflower meal, and citrus pulp) in diets for pacu (*Piaractus mesopotamicus*) and reported that soybean meal and sunflower meal can be included without impairing growth performance.

When specific growth rate and length gain were analyzed simultaneously using multivariate ANOVA (MANOVA), no significant differences were observed among treatments (Wilks’ λ = 0.948; *F* = 0.979; *p* = 0.630).

Similarly, dietary fiber levels did not affect the hematological parameters of the red blood cell series. No significant differences were observed among treatments for Ht, Hb, erythrocyte count (RBC), MCV, MCH, and MCHC (Table 3).

**Table 3:** Hematological parameters of tambaqui (*Colossoma macropomum*) juveniles fed different levels of tucumã peel (*Astrocaryum aculeatum*).

Hematological parameters	0%	3%	6%	9%	12%
Hematocrit (%)	32.1ᵃ ± 6.6	30.2ᵃ ± 5.3	31.0ᵃ ± 3.8	31.9ᵃ ± 5.5	29.3ᵃ ± 5.4
Hemoglobin (g/dL)	3.7ᵃ ± 0.8	4.0ᵃ ± 0.9	4.1ᵃ ± 0.7	3.6ᵃ ± 0.6	3.7ᵃ ± 0.9
RBC (×10^6^ mm^−3^)	2.4ᵃ ± 0.6	2.0ᵃ ± 0.6	2.3ᵃ ± 0.7	1.7ᵃ ± 0.7	2.5ᵃ ± 0.8
MCV (µm^3^)	151.8ᵇ ± 5.6	151.5ᵇ ± 4.8	151.9ᵇ ± 7.3	216.2ᵃ ± 10.5	125.6ᶜ ± 4.0
MCH (pg)	17.1ᵇ ± 5.9	20.3ᵃᵇ ± 5.9	19.5ᵃᵇ ± 8.6	24.7ᵃ ± 11.8	15.8ᵇ ± 5.4
MCHC (%)	14.0ᵃ ± 1.0	13.7ᵃ ± 0.9	13.3ᵃ ± 0.9	12.1ᵃ ± 0.9	13.8ᵃ ± 0.9

*Note:* Means followed by the same letter in the same row do not differ significantly (*p*  > 0.05; ANOVA followed by Tukey’s test).

Hematological parameters were also analyzed simultaneously using MANOVA, which confirmed the absence of significant effects (Wilks’ λ = 0.863; *F* = 1.44; *p* = 0.126). A multivariate analysis of covariance (MANCOVA) further indicated that Ht, Hb, and erythrocyte parameters were not jointly affected by fiber levels (Wilks’ λ = 0.857; *F* = 1.48; *p* = 0.107), nor by specific growth rate (Wilks’ λ = 0.965; *F* = 1.66; *p* = 0.177) or growth rate (Wilks’ λ = 0.975; *F* = 1.17; *p* = 0.322).

According to Reece [15], hematology is the study of blood cells, allowing the evaluation of morphological and quantitative changes in corpuscular elements (erythrocytes, leukocytes, and thrombocytes). Blood plasma is composed of ~90% water and 7% proteins (globulins and albumin), in addition to mineral salts, vitamins, lipids, carbohydrates, cofactors, and other circulating metabolites. Thus, hematology is an important tool for assessing the health, nutritional, and immunological status of fish and serves as a biological indicator for monitoring the overall condition and the presence of infectious agents [16].

In the present study, hematological parameters indicated that fish health was not affected by the different levels of dietary fiber from tucumã peel, and no adverse effects were observed, as evidenced by the absence of mortality. Therefore, the diets provided contained sufficient nutrients to maintain the fish’s health.

Similar results were reported by Aride et al. [17], who evaluated physiological parameters of tambaqui fed diets supplemented with the Amazonian fruit camu camu (*Myrciaria dubia*) and also observed no significant changes in hematological parameters.

The plasma biochemical profile (Table 4) of tambaqui was affected by different levels of dietary fiber from tucumã peel (*Astrocaryum aculeatum*). MANOVA revealed that plasma biochemical parameters were significantly affected by treatments (Wilks’ λ = 0.280; *F* = 5.43; *p*  < 0.001).

**Table 4:** Mean ± standard deviation of the biochemical parameters of juvenile tambaqui (*Colossoma macropomum*) fed diets containing different levels of tucumã peel (*Astrocaryum aculeatum*).

Biochemical parameters	0%	3%	6%	9%	12%
Protein (g/dL)	2.5^a^ ± 0.4	2.3^a^ ± 0.4	2.4^a^ ± 0.4	2.4^a^ ± 0.3	2.3^a^ ± 0.5
Albumin (g/dL)	1.2^a^ ± 0.3	1.2^a^ ± 0.5	1.1^ab^ ± 0.3	1.1^ab^ ± 0.3	1.1^b^ ± 0.3
Globulin (g/dL)	1.3 ± 0.3	1.2 ± 0.4	1.4 ± 0.4	1.3 ± 0.3	1.2 ± 0.5
Glucose (mg/dL)	102.1^a^ ± 10.3	92.6^b^ ± 8.6	96.9^ab^ ± 7.4	91.6^b^ ± 6.4	91.5^b^ ± 8.3
Triglycerides (mg/dL)	175.2^a^ ± 24.4	149.5^b^ ± 29.7	142.5^bc^ ± 29.8	143.4^bc^ ± 36.4	127.9^c^ ± 29.2
Cholesterol (mg/dL)	100.5^ab^ ± 10.5	94.6^b^ ± 12.2	106.4^ab^ ± 15.9	102.8^ab^ ± 10.8	101.1^ab^ ± 1.3

*Note:* Values are expressed as mean ± standard deviation. Means within the same row followed by different superscript letters differ significantly according to Tukey’s multiple comparison test (*p*  < 0.05).

ANOVA indicated that triglyceride levels (*F* = 5.86; *p*  < 0.001) and total cholesterol (*F* = 17.74; *p*  < 0.001) were significantly affected by dietary fiber levels. In contrast, plasma protein (*F* = 1.33; *p* = 0.257), albumin (*F* = 1.53; *p* = 0.189), and glucose levels (*F* = 1.92; *p* = 0.099) were not significantly affected.

MANCOVA showed that plasma biochemical variables were influenced by fiber levels (Wilks’ λ = 0.290; *F* = 5.13; *p*  < 0.001) and by specific growth rate (Wilks’ λ = 0.804; *F* = 4.24; *p* = 0.002), but not by growth rate (Wilks’ λ = 0.904; *F* = 1.85; *p* = 0.111).

Analysis of covariance (ANCOVA) revealed that plasma protein (*F* = 8.76; *p* = 0.004) and albumin (*F* = 11.41; *p* = 0.001) concentrations were significantly influenced by specific growth rate.

**Figure 1:**
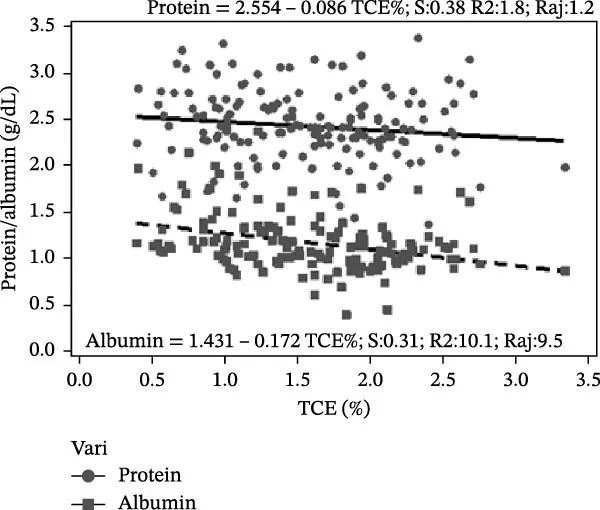
Correlation between protein and albumin with the specific growth rate of juveniles of tambaqui (*Colossoma macropomum*) fed different levels of tucumã bark.

Considering the effect of the growth rate on plasma protein and albumin concentrations, regression analysis revealed a negative linear relationship (Figure 1). Plasma protein (protein = 2.59 − 0.111 × growth rate) and albumin (albumin = 1.437 − 0.176 × growth rate) concentrations decreased as the growth rate increased in tambaqui, regardless of the dietary fiber level.

The nutritional status of fish can be assessed through plasma protein concentrations, particularly total protein and albumin, which are considered important indicators of nutritional status [18]. According to Gibbs et al. [19], low total protein values may indicate dietary deficiency, while low albumin levels may reflect protein deficiency under nonpathological conditions.

González et al. [18] also highlighted that hypoalbuminemia may be associated with malnutrition. In the present study, despite increasing dietary fiber levels, no significant effect on plasma protein or albumin concentrations was observed. However, a significant relationship between the growth rate and protein levels was identified, suggesting that the observed variation may be associated with metabolic demand due to individual growth rates rather than dietary fiber levels.

Regarding the fatty acid profile (Table 5), the main acids found were palmitic (C16:0), oleic (C18:1ω9), linoleic, and margaric. Lipids are water‐insoluble organic compounds whose main functions are energy supply and the carriage of fat‐soluble vitamins, and they are also sources of essential fatty acids [4]. According to Moraes and Almeida [20], the enhancement of a diet tends to increase lipid levels for protein reserves, improve food conversion, and decrease nitrogen excretion. Our study showed that triglyceride and cholesterol levels decreased as levels of tucumã peel (*Astrocaryum aculeatum*) increased and did not affect fish growth. This may be explained by an increase in the excretion of bile salts due to the fibers present in the diet, decreasing the precursors of cholesterol metabolism, as described by Pretto et al. [21] in their study of crambe bran in fresh or reduced forms. Antinutrients were observed in the silver catfish diet for the evaluation of total proteins, triglycerides, and cholesterol, and animals fed with crambe bran in natura presented less cholesterol, while total protein and triglycerides showed no alterations between treatments. According to Braga [22], foods with low cholesterol levels can help prevent cardiovascular diseases and dyslipidemia.

**Table 5:** Fatty acid profile by treatment of tambaqui juveniles (*Colossoma macropomum*) fed different levels of tucumã bark (*Astrocaryum aculeatum*).

Treatment	Fatty acids (%)								
	Palmitic	Linoleic	Oleic	Margaric	Caprylic	Lauric	Miristic	Vaccenic	Undecylenic
1	34.74	17.39	35.34	10.37	2.16	—	—	—	—
2	31.55	13.06	43.6	10.32	—	—	—	—	1.47
3	43.09	4.32	33.18	15.83	—	3.48	—	—	—
4	27.70	22.50	40.26	6.99	—	—	—	—	—
5	23.24	20.10	42.75	10.17	—	—	0.75	2.99	—

## 4. Conclusion

The inclusion of dietary fiber obtained from tucumã peel (*Astrocaryum aculeatum*) in diets for juvenile tambaqui can be used at levels of up to 12% without impairing the growth performance.

Although no negative effects on zootechnical performance were observed, significant alterations in biochemical parameters such as glucose, triglycerides, and albumin suggest that tucumã peel inclusion promoted metabolic modulation in tambaqui juveniles.

These results suggest that tucumã peel represents a promising alternative fiber source for aquaculture diets; however, further studies are recommended to evaluate long‐term effects and different physiological conditions.

## Funding

The funding agencies that provided financial assistance to FAPEAM Notice: Edital n. 17/2024 ‐ Divulga CT&I/Fapeam.EDITAL INOVATEC, CT‐ AGRO e Capes: PDPG – Emergencial de Consolidação Estratégico 3 e 4/Capes e PDPG – Pós Doutorado Estratégico/Capes; Projetos Fapeam: Chamada Edital n° 017/2024 – Divulga CT&I/FAPEAM.

## Conflicts of Interest

The authors declare no conflicts of interest.

## Data Availability

The data supporting the findings of this study are available from the corresponding author upon reasonable request.
